# On the Generalization of Deep Learning Models in Video Deepfake Detection

**DOI:** 10.3390/jimaging9050089

**Published:** 2023-04-29

**Authors:** Davide Alessandro Coccomini, Roberto Caldelli, Fabrizio Falchi, Claudio Gennaro

**Affiliations:** 1 Istituto di Scienza e Tecnologie dell’Informazione, 56124 Pisa, Italy; 2 National Inter-University Consortium for Telecommunications (CNIT), 50134 Florence, Italy; 3 Faculty of Economics, Universitas Mercatorum, 00186 Rome, Italy

**Keywords:** deepfake detection, deep learning, computer vision, generalization

## Abstract

The increasing use of deep learning techniques to manipulate images and videos, commonly referred to as “deepfakes”, is making it more challenging to differentiate between real and fake content, while various deepfake detection systems have been developed, they often struggle to detect deepfakes in real-world situations. In particular, these methods are often unable to effectively distinguish images or videos when these are modified using novel techniques which have not been used in the training set. In this study, we carry out an analysis of different deep learning architectures in an attempt to understand which is more capable of better generalizing the concept of deepfake. According to our results, it appears that Convolutional Neural Networks (CNNs) seem to be more capable of storing specific anomalies and thus excel in cases of datasets with a limited number of elements and manipulation methodologies. The Vision Transformer, conversely, is more effective when trained with more varied datasets, achieving more outstanding generalization capabilities than the other methods analysed. Finally, the Swin Transformer appears to be a good alternative for using an attention-based method in a more limited data regime and performs very well in cross-dataset scenarios. All the analysed architectures seem to have a different way to look at deepfakes, but since in a real-world environment the generalization capability is essential, based on the experiments carried out, the attention-based architectures seem to provide superior performances.

## 1. Introduction

Deep Learning has greatly impacted society, leading to impressive advancements in various fields. However, its use can also have negative consequences, for example, the creation of deepfakes. Deepfakes are manipulated images or videos that depict subjects in ways they never actually were, which can harm reputations or manipulate reality. Indeed, although deepfakes have numerous potential applications in the fields of entertainment, art, and education, they also pose significant security and ethical risks. For this reason, it is crucial to continue the development of robust deepfake detection methods to counteract such a threat. To tackle this problem, researchers have developed deepfake detection techniques, which are usually based on deep learning as well. These methods try to identify any traces introduced during the manipulation process, but they require large amounts of data for training. Furthermore, deepfakes are generated by resorting to different typologies of techniques and/or procedures (often even unknown) that emerge almost daily, so it is not possible to follow each methodology and consequently to re-adapt the training phase. On this basis, to have more effective deepfake detectors, researchers aim for a system that can generalize the concept of deepfakes and identify them regardless of the manipulation technique used, even if it is novel and not present in the training data. During training, a huge amount of heterogeneous data are needed to provide to the models in order for them to see enough forms of deepfakes to stimulate them to abstract and generalize. In this research, a comparison was made among different deep learning architectures in order to validate their generalization capabilities, specifically against deepfake videos. It is worth saying that techniques used to manipulate videos do not necessarily introduce the same anomalies and features that can be embedded when tampering with still images. It is therefore interesting to show how different models behave in this context and how they look at the video to understand if it has been manipulated. In particular, we compared three different kinds of network architectures: a convolutional network, such as *EfficientNet V2,* a standard *Vision Transformer*, and also a *Swin Transformer* which is a specific type of transformer inspired to use Convolutional Neural Networks’ hierarchical approach. Our experiments indicate that the Vision Transformer outperforms other models in terms of generalization ability when evaluated in a cross-forgery context, while the Swin Transformer seems to be better in the cross-dataset experiments. This probably stems from the attention mechanism which enables the model to abstract better the concept of deepfake, but only with the constraint of availability of a large quantity and diversity of data used during training. On the other hand, the Vision Transformer struggles to learn when data are limited, unlike the EfficientNet-V2 and Swin Transformer, which perform satisfactorily even under such constraints.

## 2. Related Works

### 2.1. Deepfake Generation

Deepfake generation techniques refer to the methods used to manipulate a human face, changing its appearance or identity in a realistic manner. There are two main categories of approaches: those based on Variational AutoEncoders (VAEs) [1] and those based on Generative Adversarial Networks (GANs) [2]. VAE-based methods use encoder–decoder pairs to decompose and recompose two distinct faces. By swapping the decoders, it is possible to transform one face into the other, resulting in a credible output. GAN-based methods, on the other hand, use two different networks: a discriminator, trained to classify whether an image is fake or real, and a generator that generates a fake face to fool the discriminator. This results in a feedback loop, where the generator is trained to improve its performance based on the output of the discriminator. GANs are typically more powerful than VAEs, but also more challenging to train. Some of the most popular GAN-based deepfake generation methods include Face2Face [3] and FaceSwap [4]. Recently, a number of other deepfake generation approaches have been proposed, leveraging the advancements in computer vision and deep learning. For example, reference [5] presents a method for synthesizing realistic talking heads from a single source video. Reference [6] proposes StyleGAN, a highly-customizable deepfake generation method that allows for fine-grained control over the generated images.

### 2.2. Deepfake Detection

As deepfake generation methods become increasingly sophisticated, there is a growing need for systems that can distinguish between real and manipulated images. This is a problem not just in the field of images, but also in text, where recent work such as [7] has analysed deepfakes in tweets to identify fake content on social networks. To tackle the challenge of deepfake detection in videos, many video-based deepfake detectors have been developed. Even if some approaches propose solutions which also analyse the temporal information of manipulated videos [8,9,10,11], the majority of methods are frame-based, classifying each video frame individually. Furthermore, several competitions have been organized to stimulate the resolution of this task including [12,13]. To train effective deep learning models for deepfake detection, numerous datasets have been created over the years, including DF-TIMIT [14], UADFC [15], FaceForensics++ [16], Celeb-DF [17], Google Deepfake Detection Dataset [18], DFDC [12], Deepforensics [19], and ForgeryNet [20]. The latter dataset, which is the most complete, large, and diverse, has recently emerged as a popular choice for deepfake detection research. One type of Convolutional Neural Network, EfficientNet, has emerged as particularly effective in solving the task, and is the basis of many state-of-the-art solutions, such as the winning solution of the deepfake detection challenge [21]. More recently, with the rise of Vision Transformers in Computer Vision, new deepfake detection solutions have been developed, such as the method in [22] which combines Transformers with convolutional networks to extract patches from faces, and the approach in [23], which uses a pretrained EfficientNet B7 with a Vision Transformer, trained through distillation. An innovative work on combining different types of Vision Transformers, such as the Cross Vision Transformer [24] and EfficientNet B0, is presented in [25]. EfficientNet has been further improved with the introduction of EfficientNetV2 [26], a version that is optimized for smaller models, faster training, and better ImageNet performance. An evolution of this approach which combines together a TimeSformer and a Convolutional Neural Network is presented in [8], where various deepfake detection problems such as multi-identity and face size variation are also treated.

## 3. The Followed Approach and the Tested Network Architectures

To validate the neural network’s ability to detect deepfakes generated by methods not used in its training set, a dataset containing a variety of deepfake generation methods and labels is needed. The chosen dataset for this purpose is ForgeryNet [20], which is one of the most comprehensive deepfake datasets available, containing 2.9 million images and 220,000 video clips. The fake images are manipulated using 15 different manipulations while the videos are manipulated using only 8 of them [27,28,29,30,31,32,33,34,35,36]. To each image and video, more than 36 mix-perturbations are randomly applied on more than 4300 distinct subjects. Examples of applied perturbations are optical distortion, multiplicative noise, random compression, blur, and many others shown in more detail in the ForgeryNet paper [20]. Furthermore, the different manipulations applied can be grouped into two macro-categories, *ID-Remained* and *ID-Replaced*. The first category involves manipulations of the subject’s face without changing their identity, while the second category involves replacing the subject’s face with a different one. These two categories are further divided into four sub-categories: all the videos falling under the ID-Remained category are manipulated with Face Reenactment methods, while the ID-Replaced class is divided into Face Transfer, Face Swap, and Face Stacked Manipulation (FSM). These sub-categories collectively make up a significant portion of the deepfake generation techniques currently known. The ForgeryNet dataset includes people in various settings and situations.

The extracted frames are pre-processed, similar to many other deepfake detection methods [8,9,10,22,25] by introducing a face extraction step using the state-of-the-art face detector, MTCNN [37]. The models are trained and evaluated on a per-face basis and data augmentation was performed, similar to [8,21,25]. However, we extracted the faces to be squared and with an additional 30% padding in order to also catch a portion of the background behind the person. We exploited the Albumentations library [38] and applied common transformations randomly during training. Whenever an image is an input to the network during training, it is randomly resized using three types of isotropic resize with different interpolation methods (area, cubic, or linear). Afterwards, random transformations such as image compression, gaussian noise, horizontal flip, brightness or saturation distortion, grayscale conversion, shift, rotation, or scaling, are applied.

The present paper is derived from another work [39] where we already conducted a similar cross-forgery analysis on the part of the dataset consisting of still images. In this case, we performed our analysis on videos and, in particular, we have made a specific comparison among different kinds of architectures. It is important to carry out this analysis on videos because the anomalies that are introduced in videos can also differ greatly from those that may result from the manipulation of a single image. Therefore, the behaviour of the various deep learning methods can also change greatly. In the ForgeryNet dataset, there is a label assigned to each video indicating whether it has been manipulated or not. Additionally, the label specifies the method employed to perform the manipulation. Among the methods used, FaceShifter and ATVG-Net manipulate all frames of the video, while the other methods partially manipulate the video frames, providing information on which frames have been manipulated and which ones are left unaltered.

To perform this comparative analysis on cross-forgery generalization capability we have considered three kinds of network architectures. Convolutional Neural Networks (CNNs), a widely used type of neural network in computer vision, and two Vision Transformers (ViTs) [40], a newer, highly competitive deep learning model. As the representative of the CNN category, we have taken an *EfficientNetV2-M* [26], which is a newer and more advanced version of the well-known EfficientNet. EfficientNets are widely used in deepfake detection and remain a cornerstone of many state-of-the-art methods on leading datasets. In contrast, for one of the Vision Transformers, we have used the *ViT-Base*, a ViT of similar dimensions to the CNN, which was one of the first versions introduced. Additionally, a third architecture, a Swin Transformer [41], has been taken into account; this has been included because this type of Transformer is particularly interesting for our analysis in that although it is attention-based, the computation of attention takes place in a hierarchical manner emulating the convolutional layers of CNNs. The Swin Transformer is an architecture for image classification that improves the traditional transformer approach by using hierarchical feature representations and a window-based attention mechanism. It divides the input image into patches and transforms them into low-dimensional feature vectors using a learnable projection. These vectors are then passed through a series of transformer blocks, and organized into stages to capture spatial and channel-wise dependencies. Finally, the output is passed through a classification head to produce the class probabilities. The Swin Transformer achieves state-of-the-art performance while being computationally efficient and scalable to larger image sizes. The Swin Transformer selected for our experiments is the *Swin-Small*. All the models were pretrained on ImageNet-21k and fine-tuned on sub-datasets from ForgeryNet, which were constructed with a nearly equal balance of fake and real images as explained in the next section. To reduce false detections, only faces with a confidence level above 95% were included. All networks were trained by freezing a number of layers such that the trained parameters correspond to approximately 45 M. In particular, only the last layers of the models considered were made trainable so that the number of parameters was always comparable between the various experiments, while the other layers’ weights remained at the values based on the pretraining.

## 4. Experiments

### 4.1. General Setup

The experiments conducted in our research are divided into two parts. In the first part, we used frames from pristine videos and manipulated frames from fake videos generated with one deepfake generation method at a time to compose a training set. Each obtained model is then tested against frames extracted from videos manipulated with the same generation methodology used at training time but also against other methods not seen during the training phase in order to investigate the generalization capacity of the different architectures. The classification task is always conducted frame-by-frame. In the second part, we used multiple deepfake generation methods grouped by category (ID-Replaced or ID-Remained) to construct the training set. Since the labels of the ForgeryNet test set were not available at the time of the experiments, we used the validation set, which we will refer to as the test set, for all evaluations. During training, a 10% portion, consistent for all models, was randomly selected from the training set and referred to as the validation set. The models were trained for up to 50 epochs with a patience of 5 epochs on the validation set, using the Binary Cross Entropy Loss (BCE) and an SGD optimizer with a learning rate of 0.1 that decreases with a step size of 15 and a gamma of 0.1.

### 4.2. Single Method Training

**Figure 1:**
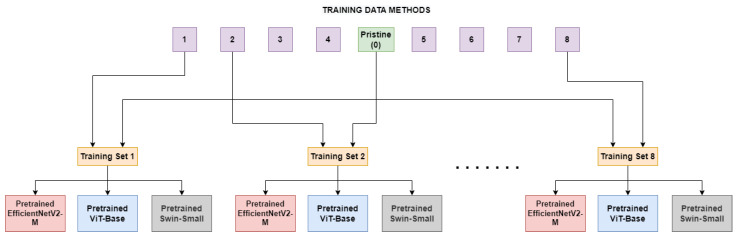
The *Single Method Training* setup: eight different training sets are constructed, each consists of frames manipulated with a deepfake generation method and pristine frames.

In this section, we outline the process used to examine a model’s ability to recognize images manipulated by various deepfake generation methods, despite being trained on real images and images manipulated with only one deepfake method. In the first comparison, the three models under consideration, namely EfficientNetV2-M, ViT-Base, and Swin-Small, were fine-tuned on each of the eight sub-datasets as illustrated in Figure 1. These sub-datasets consisted of both unaltered frames and frames manipulated using specific techniques, specifically FaceShifter(1), FS-GAN(2), DeepFakes(3), BlendFace(4), MMReplacement(5), DeepFakes-StarGAN-Stack(6), Talking-Head Video(7), and ATVG-Net(8). As displayed in Table 1, the sizes of the datasets vary quite largely. Pristine frames are most common within the dataset so, to ensure a good balance without sacrificing too many of them, a subset equal to the number of fake frames of the method under training is randomly selected at each epoch. In this experiment, the models will only encounter, during the training, anomalies generated by one specific deepfake method at a time. This may cause a tendency in the models to learn that a video is manipulated only when some specific artifacts occur, causing a lack of generalization. To validate this and discover architectures’ limitations, the models trained on the sub-datasets were then tested on frames in the test set, including those manipulated by methods not used during training.

### 4.3. Multiple Methods Training

**Figure 2:**
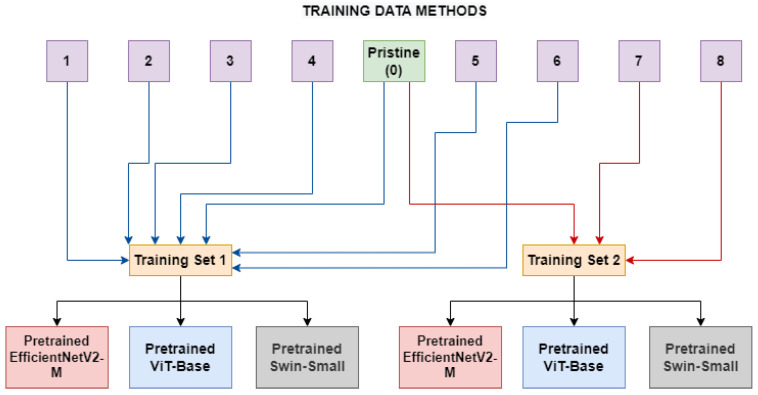
The *Multiple Method Training* setup: two different training sets are constructed, each consists of frames manipulated with deepfake generation methods related to the same category (blue lines for *ID-Replaced* and red lines for *ID-Remained*) and pristine frames.

A second experiment has been conducted by training the models on real frames and frames manipulated using a group of methods belonging to the same category (*ID-Replaced* or *ID-Remained*), as shown in Figure 2. This was examined to determine if the networks can better generalize in the presence of diverse categories of manipulation methods, which may introduce a greater variety of artifacts. Hopefully, the models trained in this setup will need to abstract the concept of deepfake to a level which is not highly related to the seen artifacts.

In Table 2, the sizes, in terms of available frames, of the two different categories can be seen. As depicted in Figure 2, two models (for each network architecture) have been trained: the first one (*ID-Replaced*) is based on frames crafted by using methods belonging to the ID-Replaced category (methods from 1 to 6), while the second one (*ID-Remained*) is based on those ones coming from the ID-Remained category (methods 7 and 8). In both of them, also pristine images from unaltered videos are added to the training dataset.

## 5. Results

### 5.1. Single Method Training

**Figure 3:**
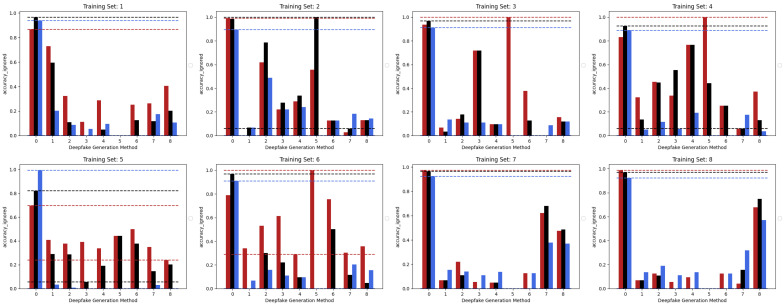
The performance in terms of accuracy achieved by each of the three considered models with respect to the eight different training sets following the *Single Method Training* setup: EfficientNetV2-M (red), Swin-Small (black), and ViT-Base (blue).

Figure 3 shows the accuracies achieved by the three considered models trained in the *Single Method Training* setup, presented in the previous section, with respect to each of the methods comprised within the test set. Looking at the accuracies of the three models, it can be pointed out that the EfficientNetV2-M and the Swin-Small maintain results often above 80% in correspondence of test frames manipulated with the same methods used in the training set (as expected) and, at the same time, obtain a certain degree of generalization. In fact, the same models sometimes succeed in detecting frames manipulated with methods unseen during training, although only reaching values of accuracy that are quite limited. The case of method number 5 (*MMReplacement*) is rather anomalous, though the detection percentage is often very high indeed; this behaviour is probably induced by the low number of available examples (see Table 1).

**Figure 4:**
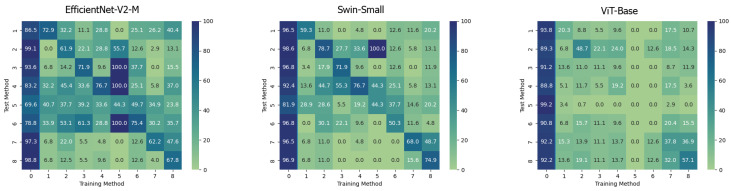
Confusion matrices of the frame-level accuracy values for the three models under consideration trained in the *Single Method Training* setup and tested on frames manipulated with all the available methods, respectively.

On the contrary, it can be easily noticed that, in all the cases, the ViT-Base is substantially unable to learn in the presence of relatively few training images. In fact, for instance, by training the model on methods 3, 4, 5, and 6 and then testing it on the test set, it is evident that the model is substantially underfitting and practically unusable compared to the two others taken into consideration. Interestingly, the Swin Transformer, although also based on the attention mechanism, is not particularly affected by this phenomenon and instead succeeds in obtaining competitive results in all contexts. This probably lies in the hierarchical nature that emulates the convolutional layers of traditional CNNs and thus allows it to exploit implicit inductive biases better. Good performances are preserved, in any case, with respect to pristine frame detection. In this setup, the architecture based on a convolutional network seems to prove more capable of generalization. The accuracies obtained from the three models are also shown in the confusion matrices in Figure 4 where all previously commented trends are reconfirmed again.

### 5.2. Multiple Methods Training

**Figure 5:**
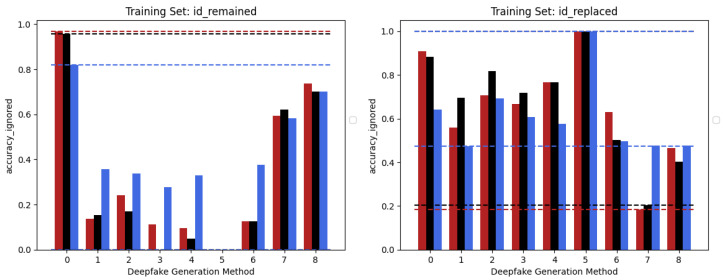
Accuracy performances achieved by each of the models considered in the two different training sets constructed following the *Multiple Methods Training* setup: EfficientNetV2-M (red), Swin-Small (black), and ViT-Base (blue). *ID-Replaced* methods (1–6), *ID-Remained* methods (7–8), and *Pristine* (0).

**Figure 6:**
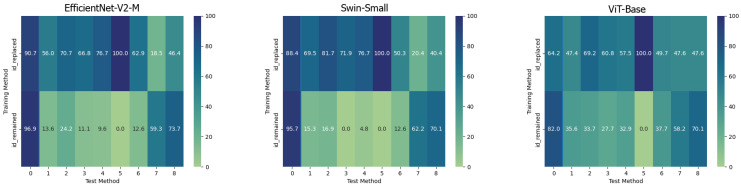
Confusion matrices of the frame-level accuracy of the three models trained in the *Multiple Method Training* setup and tested on frames manipulated with all available methods.

The behaviour of the three networks is now analysed in the second considered setup, namely *Multiple Methods Training*, and corresponding results are shown in Figure 5. In this case, the datasets are composed of frames extracted from videos manipulated by not only one method, so the models will have more difficulty focussing on specific artifacts and be forced to generalize. In this setup, the situation is significantly different from the previous one. Surprisingly, the classic Vision Transformer, which previously struggled to train effectively, is now the only model capable of generalizing well to frames that have been manipulated using techniques that were not present in the training data. This result probably stems from the fact that the training set consists of significantly more images than in the previous setup and it is strongly in line with what is presented in [39]. This particular architecture shows in many contexts a major need for data and resources which, when available, enable it to achieve very competitive results. In this case, the confusion matrices (see Figure 6) clearly show the greater generalization capacity of the Vision Transformer although at the expense of more false positives. In fact, the “pristine” class is less accurately classified by this latter. This may be a problem since in a real-world context we may want to reduce as much as possible the number of false alarms, in particular, if the system is fully automated.

### 5.3. Cross-Dataset Evaluation

To further evaluate the generalization capability of the trained models we also tested them in a cross-dataset context. In particular, we considered the three architectures trained on videos manipulated with ID-Remained or with ID-Replaced methods (ForgeryNet dataset) and tested on the well-known DFDC Preview test set. In Table 3, we report the AUC values of the trained models compared with previous works in the literature. This is probably the most challenging scenario since both the contexts and the manipulation methods are significantly different, and indeed the performances of the models are pretty low. In particular, the EfficientNets are totally incapable of detecting these deepfakes with a very low AUC value. On the other hand, attention-based methods manifest better performances even if, as expected, they are worse than other, more complicated and articulated, methods in the literature. The Swin-Small trained on the videos manipulated with ID-Replaced methods perform pretty well with an AUC of 71.2%, demonstrating a good level of generalization. Again, also in this context, it seems that attention may be the key to achieve better generalization performances while the considered CNN is in any case too tied with the methods seen during training. Furthermore, the trained models which achieve better performances are the ones trained on a more complete and heterogeneous dataset, namely the videos manipulated with ID-Replaced methods, highlighting again the need for these architectures for a huge amount of data.

Despite the limited amount of data and variety available in the setup presented in Section 4.2, we conducted a cross-dataset test with the models trained in this manner too. The results illustrated in Table 4 confirm the previous findings, and thus show the difficulty on the part of all models to generalize in cross-dataset contexts, with a slight superiority of the Swin Transformer.

## 6. Conclusions

In this study, we investigated the generalization capabilities for detecting deepfake videos of various deep learning architectures by using two different setups. The first setup involved a limited training set constructed from both pristine and manipulated video frames adopting a specific method at a time and then testing versus all the different methods. In this setup, the EfficientNet-V2 convolutional network outperformed the Vision Transformer in learning from the less diverse and limited training data, while the Swin Transformer showed promising results. In the second setup, we considered a larger and more variegated training dataset that included frames coming from deepfake videos on which have been applied different manipulation methods, but these are belonging to the same category (*ID-Replaced* or *ID-Remained*), and then performing cross-testing. Interestingly, the Vision Transformer demonstrated superior generalization capabilities and outperformed the convolutional network in detecting frames from videos manipulated with novel methods. This result is tied to higher resource availability, both in data and computational terms, which is not always possible to achieve.

Our findings suggest that in real-world scenarios where large, diverse deepfake detection datasets are available and generalization is critical, the Vision Transformer may be the optimal choice for detecting deepfakes. However, in cases where the training data are limited, a convolutional network such as EfficientNet-V2 may be more suitable and be considered a good enough alternative. The Swin Transformer provides a good balance between the two in terms of generalization and performance demonstrating a good generalization capability in all the considered contexts and a pretty low false-positive rate. It also results in being significantly more capable of generalizing the concept of deepfake when tested in a cross-dataset scenario. This suggests that probably the attention mechanisms may enable the models to better generalize the concept of deepfakes but only when enough data are provided.

Overall, our study highlights the significance of considering the specific characteristics of the dataset and deep learning architecture when detecting deepfakes to be able to create a detector which may be applied in the real world.

## Figures and Tables

**Table 1:** Number of frames for Single Methods Training and Test setup.

Video Manipulation Methods	Training Frames	Test Frames
0 (*Pristine*)	118,255	47,369
1 (FaceShifter)	13,337	1889
2 (FS-GAN)	48,122	8732
3 (DeepFakes)	8550	1157
4 (BlendFace)	9827	1335
5 (MMReplacement)	270	115
6 (DeepFakes-StarGAN-Stack)	3610	509
7 (Talking-Head Video)	26,338	2199
8 (ATVG-Net)	37,449	5383

**Table 2:** Number of frames for Multiple Methods Training and Test.

Video Manipulation Categories	Training Frames	Test Frames
0 (*Pristine*)	118,255	47,369
ID-Replaced	83,716	13,737
ID-Remained	63,787	7582

**Table 3:** Cross-dataset comparison of video-level AUC on the DFDC Preview test set.

Model	Train Set	AUC
Face X-ray [42]	FF++	65.5
Patch-based [43]	FF++	65.6
DSP-FWA [44]	FF++	67.3
CSN [45]	FF++	68.1
Multi-Task [46]	FF++	68.1
CNN-GRU [47]	FF++	68.9
Xception [48]	FF++	70.9
CNN-aug [49]	FF++	72.1
LipForensics [50]	FF++	73.5
FTCN [10]	FF++	74.0
RealForensics [45]	FF++	75.9
RealForensics [45]	FF++	75.9
iCaps-Dfake [51]	FF++	76.8
MINTIME-XC [8]	ForgeryNet (All)	77.9
EfficientNet-V2-M	ForgeryNet (ID-Remained)	50.0
	ForgeryNet (ID-Replaced)	50.1
ViT-Base	ForgeryNet (ID-Remained)	51.0
	ForgeryNet (ID-Replaced)	57.2
Swin-Small	ForgeryNet (ID-Remained)	58.7
	ForgeryNet (ID-Replaced)	71.2

**Table 4:** Cross-dataset in depth analysis: the architectures are trained on each of the 8 kinds of ForgeryNet deepfake manipulations and then tested on the DFDC Preview test set. AUC is given accordingly.

Model	Train Set (ForgeryNet)	AUC
EfficientNet-V2-M	Method 1	51.0
	Method 2	50.3
	Method 3	47.0
	Method 4	49.7
	Method 5	50.3
	Method 6	47.0
	Method 7	52.5
	Method 8	50.0
ViT-Base	Method 1	53.3
	Method 2	52.5
	Method 3	43.0
	Method 4	52.0
	Method 5	52.3
	Method 6	51.3
	Method 7	50.5
	Method 8	49.8
Swin-Small	Method 1	53.0
	Method 2	65.3
	Method 3	58.0
	Method 4	59.5
	Method 5	58.0
	Method 6	55.3
	Method 7	59.3
	Method 8	56.7

## Data Availability

Not applicable.
